# Editorial: The Role of Peptide Hormones in Insect Physiology, Biochemistry, and Molecular Biology Processes, Volume II

**DOI:** 10.3389/fphys.2021.815010

**Published:** 2021-12-09

**Authors:** Klaus H. Hoffmann, Dov Borovsky, Yonggyun Kim

**Affiliations:** ^1^Department of Animal Ecology I, University of Bayreuth Animal Ecology I, Bayreuth, Germany; ^2^Department of Biochemistry and Molecular Genetics, University of Colorado Anschutz Medical School, Aurora, CO, United States; ^3^Department of Plant Medicals, Andong National University, Andong, South Korea

**Keywords:** CAPA-PVK neuropeptide and the immune system, insulin like peptide and growth factor, CRISPER/Cas9 deletion of insulin like peptides, ecdysis triggering hormone receptors, bursicon, control of trypsin translation, control of trypsin translation with TMOF, nutrients and neuropeptide expression

The study on the effect of short-term desiccation and the role of CAPA-PVK neuropeptide on the immune system of *Nicrophorus vespilloides* by Urbanski et al. showed that environmental conditions play a major role in the immune system. Short-term desiccation and Tenmo-PVK-2, a member of the CAPA-PVK neuropeptide family, effects on the immune system were investigated. Short-term desiccation decreased the phagocytic activity of the hemocytes and their adhesive ability. A significant increase in phenoloxidase (PO) and *proPO* expression levels were observed indicating that melanin deposition prevented water loss. The elevated expression of *defensin* is due to environmental stress and pathogen infection. A recovery time of 1 h restored to normal cellular and humoral mechanisms except for inhibited PO activity and down regulation of *proPO*, confirming that melanin deposition plays an important role in preventing water loss. Similarly, the application of Tenmo-PVK2 indicated that the CAPA-PVK neuropeptides also influence the immune system.

Chowanski et al. reported in a review article that insulin-like peptide (ILP) and insulin-like growth factor (IGF) signaling pathways play crucial roles in regulatory functions such as metabolism, growth and development, fecundity, stress resistance, and lifespan. ILP are encoded by multiple gene families in the nervous and non-nervous organs. The peptides interact with different factors like hormones, neurotransmitters, and growth factors. The authors indicate that three interactions appear to be most significant (1) combined influence of ILP and other factors on the same target cells, (2) effect of ILP on synthesis/secretion of other factors that regulate metabolism, and (3) regulating of the activity of cells that produce or secrete ILP by other factors. An overview of the regulatory role of ILPs in insect metabolism and their interaction with other factors that control metabolism is presented.

Al Baki et al. used CRISPER/Cas9 produced deletion mutants of two insulin-like peptides (ILPs) in *Maruca vitrata* to investigate whether all ILPs are needed for development and reproduction. Two ILPs (ILP1 and ILP2) mutants of *M. vitrata* that mediate sugar level, larval development, and adult reproduction were generated. Although the mutated insects developed into adults, they produced high levels of trehalose titer in the hemolymph and the hyperglycemic effect was more pronounced in the ILP2 mutant. Both mutants showed high expression of *trehalose-6-phosphate synthase* and suppressed expression of *trehalase*. The expression levels of the insulin receptor and *Akt* genes were upregulated, whereas *FOXO* and *TOR* genes were downregulated. These mutants developed slow, exhibited reduced body size, with poor oocyte development, poor *vitellogenin* gene expression and males showed impaired reproductive activities. Therefore, both ILPs are required for larval development and adult reproduction.

The functional characterization of Ecdysis Triggering Hormone Receptors (AgETHR-A and AgETHR-B) in *Anopheles gambiae* was reported by Jindal et al. These authors using heterologous expression system in, mammalian cell line, manually annotated the ETH receptor gene (AGAP002881). Two splicing variants of the ecdysis triggering hormone receptors A and B were detected and the manual annotation allowed N-terminal and exon-intron boundaries determination of the gene. Using a calcium mobilization assay the authors reported that AgETHR-B showed 28 -fold higher sensitivity to AgETH-1 and 320-fold higher sensitivity to AgETH-2 when comparted with AgETHR-A. The receptors recognized only ETH peptides. On the other hand, ecdysis triggering hormone receptor B is more promiscuous responding to different ETH variants.

The role of bursicon, a neuropeptide belonging to the cystine knot family, in *Tribolium castaneum* was reported by Li et al. Bursicon can form heterodimers or homodimers during cuticle sclerotization, innate immunity, and midgut stem cell proliferation. Knocking down the two cysteine knot subunits (burs, pburs) and its receptor *T. castaneum rickets* (*Tcrk*) by RNAi down regulated the expression levels of Vg1 (vitellogenin 1), Vg2, and Vg receptor (VgR) in females 3 and 5 days after adult emergence causing abnormal oocytes with low Vg content. Silencing of burs reduced the number of laid eggs and prevented hatching, whereas silencing of pburs decreased the number of eggs laid, hatching rate, and larval size. The knockdown of burs or pburs downregulated the expression of insulin/insulin-like signaling TOR signaling genes encoding insulin receptor (InR), protein kinase B (Akt), and ribosomal protein S6 kinase (S6K). Injecting recombinant pburs restored *Vg and VgR*. In females with knocked down Tcrk the *Vg* and its receptor *VgR* could not be restored. Therefore, bursicon affects *Vg* expression via the Tcrk receptor and juvenile hormone (JH) IIS/TOR pathway.

The role of Trypsin Modulating Oostatic Factor (TMOF) in the control of trypsin biosynthesis in the mosquito *Culex quinquefasciatus* was reported by Borovsky et al. These authors purified the late trypsin from blood fed female mosquitoes and sequenced the N-terminal of the enzyme. The late trypsin cDNA (855 bp) was sequenced and 3D models of the pro and activated enzymes were built. The role of TMOF in controlling *Cx. quinquefasciatus* late trypsin was studied by injecting TMOF into females immediately after blood feeding, showing that the translation of the trypsin's transcript was inhibited but not the synthesis of the transcript. These results suggest that the physiological role of TMOF is to control the translation of trypsin in female *Cx. quinquefasciatus* during blood digestion.

The role of nutrients and neuropeptide expression in *Spodoptera littoralis* was studied by Dogan et al. These authors showed that energy homeostasis is controlled by the neuroendocrine system. Disruption of the energy homeostasis is implicated in cardiac dysfunction, obesity, diabetes, and fatty acid syndrome. To date, most studies use *Drosophila melanogaster* as the sole model to study the effect of different diets on insect development. Using *S. littoralis* and different diets ranging from high fat, high sugar, calcium-rich, and plant-based diet the energy homeostasis in third and fifth instar larvae was followed. Weight gain, life span, triglyceride levels, pupal development, and larval development were followed. Neuropeptide genes *SpoliAKH* and *SpoliILP1-2* expression after feeding different diets were followed in third and fifth instar larvae. No differences were found in the third instar larvae; however, some differences were observed in the 5th instar larvae showing that different diets can down regulate and upregulate these peptide hormone genes.

## Author Contributions

All authors listed have made a substantial, direct, and intellectual contribution to the work and approved it for publication.

## Conflict of Interest

The authors declare that the research was conducted in the absence of any commercial or financial relationships that could be construed as a potential conflict of interest.

## Publisher's Note

All claims expressed in this article are solely those of the authors and do not necessarily represent those of their affiliated organizations, or those of the publisher, the editors and the reviewers. Any product that may be evaluated in this article, or claim that may be made by its manufacturer, is not guaranteed or endorsed by the publisher.

